# Genome-wide identification and analysis of expression patterns of the *ABC1K* gene family members in *Medicago sativa*

**DOI:** 10.3389/fpls.2024.1486525

**Published:** 2024-11-25

**Authors:** Zhengqiang Chen, Fangqi Chen, Yaxuan Qin, Le Wang, Ruifang Jia, Jun Zhao, Kejian Lin, Yuanyuan Zhang

**Affiliations:** ^1^ Key Laboratory of Biohazard Monitoring, Green Prevention and Control for Artificial Grassland, Ministry of Agriculture and Rural Affairs, Institute of Grassland Research of Chinese Academy of Agricultural Sciences, Hohhot, China; ^2^ College of Horticulture and Plant Protection, Inner Mongolia Agricultural University, Hohhot, China

**Keywords:** ABC1K, alfalfa, phylogenetics, expression analysis, abiotic stress

## Abstract

The ABC1K (activity of bc1 complex kinase) atypical protein kinase family regulates diverse physiological functions in plants, including the development, growth, and response of plants to various stress stimuli. However, to date, only a few members of the alfalfa (*Medicago sativa*) ABC1K gene family have been identified, which severely limits the exploration of the functional mechanism of alfalfa *ABC1K*. Here, we identified 22 *ABC1K* genes from the alfalfa genome and categorized them into four types on the basis of phylogenetic analysis results and gene structure. We then characterized the physical and biochemical properties, chromosomal location, subcellular localization, *cis*-regulatory elements, and conserved motifs of these genes. Transcript profiling analysis confirmed that *MsABC1K*s were widely expressed in various alfalfa tissues, with tissue-specific expression. We also found that salt and drought conditions significantly regulated *MsABC1K* gene expression, thus indicating that *MsABC1K* genes perform critical functions in alfalfa’s response to abiotic stress. In summary, the findings of our study serve as an important basis to enhance the stress resistance of alfalfa and provide valuable insights to better comprehend the functions of the *MsABC1K* gene family.

## 1 Introduction

The ABC1K (activity of bc1 complex kinase) family consists of atypical protein kinases that occur in archaea, bacteria, and eukaryotes (Leonard et al., 1998). ABC1/COQ8 (also known as ScCOQ8) is the founding member of this family; in *Saccharomyces cerevisiae*, this protein regulates mitochondrial bc1 complex activity by controlling coenzyme Q (CoQ) synthesis (Bousquet et al., 1991). Subsequently, *Escherichia coli* UbiB and *Providencia stuartii* AarF were found as homologs of ScCOQ8, which also participate in CoQ biosynthesis (Poon et al., 2000; Mollet et al., 2008). The ABC1K family in *Arabidopsis* comprises 17 members; among these, eight members (AtABC1K1–8) are included in the photosynthetic-specific clade, three members (AtABC1K9, AtABC1K10a, and AtABC1K10b) belong to the ancestral clade, and six members (AtABC1K11–15) are included in the mitochondrial clade (Lundquist et al., 2012a, b; Qin et al., 2020). The ABC1K family members are also detected in other plants such as maize (Lundquist et al., 2012a), rice (Yang et al., 2012), tomatoes (Li et al., 2015b), and wheat (Gao et al., 2022). According to previous studies, most plant ABC1K proteins are localized in the chloroplast and mitochondria (Gao et al., 2015).

ABC1Ks regulate diverse physiological processes in plants. For example, in *Arabidopsis*, six ABC1K members are localized on plastoglobules inside the chloroplast; these members may phosphorylate other proteins present in plastoglobules and modulate their activity (Lundquist et al., 2012a). ABC1K7 and ABC1K8 present in *Arabidopsis* control lipid accumulation or synthesis in the chloroplast and modulate the composition of the chloroplast membrane during stress response (Manara et al., 2015). OsAGSW1, the ABC1K member in rice, has a crucial function in the shape and size modification of rice seeds by monitoring external parenchymal cell number and vascular bundle development (Li et al., 2015a). Both *Arabidopsis ABC1K7* and *ABC1K8* genes showed increased expression following treatment with abscisic acid (ABA), and *abc1k7* and *abc1k8* mutants exhibited alterations in some ABA-responsive physiological processes, thus suggesting the involvement of ABC1K7 and ABC1K8 in the interaction between ABA and reactive oxygen species (ROS) signaling (Manara et al., 2016). Defects of several mitochondria-localized *ABC1K* genes, namely, *ABC1K10a*, *ABC1K11*, *ABC1K13*, and *ABC1K15*, lead to salt hypersensitivity (Qin et al., 2020). In algal species, ABC1Ks are critical functional and structural components of plastoglobules (Lohscheider and Bártulos, 2016). ABC1K1, ABC1K3, and ABC1K8, which are plastid clade members, exert a crucial influence on oxidative stress response (Martinis et al., 2013, 2014).

Alfalfa (*Medicago sativa*), popularly called the “king of forage grasses”, is an important legume forage grass species worldwide, and it has a high yield, good palatability, and a high nutritional value (Bora and Sharma, 2011; Hrbáčková et al., 2020; Chen et al., 2021). The genome assembly files of XinJiangDaYe, an alfalfa cultivar, were published in 2019 (Chen H. et al., 2020). The XinJiangDaYe genome contains 32 allelic chromosomes assigned to eight homologous groups with four allelic chromosomes in each group, designated chr1.1, chr1.2, chr1.3, and chr1.4 to chr8.1, chr8.2, chr8.3, and chr8.4. Several alfalfa genes have been reported to date, and their functions in response to biotic and abiotic stress factors have been studied (Li et al., 2023; Liu H. et al., 2023; Sheng et al., 2022; Zhang et al., 2023); this may promote the molecular breeding of cultivated alfalfa. However, systematic identification and expression patterns of the alfalfa *ABC1K* gene family remain undetermined. This study first identified 22 alfalfa *ABC1K* genes (MsABC1Ks) and then conducted a phylogenetic analysis as well as an analysis of gene structure, composition of motifs, chromosome localization, and expression patterns of *MsABC1K*s. The results of this study can facilitate better comprehension of the potential regulatory roles of *MsABC1K* family members in controlling growth and abiotic stress response in alfalfa. The present study also provides a theoretical basis to examine the function of alfalfa *ABC1K* gene family members.

## 2 Materials and methods

### 2.1 Identification of MsABC1Ks in the alfalfa genome

XinJiangDaYe genome assembly files (https://figshare.com/projects/whole_genome_sequencing_and_assembly_of_Medicago_sativa/66380) were used to derive the genome sequence information of alfalfa. The hidden Markov model (HMM) data of the ABC1K domain (PF03109) in the PFAM database (http://pfam.xfam.org/) were acquired to identify *MsABC1K* gene family members. The HMMER 3.0 software was utilized to retrieve MsABC1K protein sequences. The identified MsABC1Ks were uploaded to the National Center for Biotechnology Information (NCBI) Conserved Domain Database website (https://www.ncbi.nlm.nih.gov/cdd) to confirm the presence of conserved structural domains.

### 2.2 Location of chromosomes and gene-related information

Based on genome annotation files, the TBtools software was utilized to visualize the chromosomal localization of the *MsABC1K* gene (Chen et al., 2020). pI values, molecular weight (MW) values, cDNA sequence length, the grand average of hydropathicity (GRAVY), and protein length of MsABC1Ks were predicted using ExPaSy online tools (https://prosite.expasy.org/) (Artimo et al., 2012).

### 2.3 Phylogenetic analysis

The neighbor-joining method along with 1,000 bootstrap replicates was utilized to generate a phylogenetic tree in the MEGA software (Kumar et al., 2018). Sequences of 17 AtABC1K proteins were collected from *Arabidopsis* genome assembly TAIR10 (http://www.arabidopsis.org/). Subsequently, rice genome assembly v6 (http://rice.plantbiology.msu.edu/) was used to acquire the protein sequences of 16 OsABC1Ks. Next, maize genome assembly 5b.60 (http://www.maizesequence.org/) was utilized to collect the protein sequences of 19 ZmABC1Ks.

### 2.4 Determination of *cis*-regulatory elements, conserved motifs, and gene structure of MsABC1Ks in alfalfa

Multiple Expectation Maximization for Motif Elicitation (MEME) suite (http://meme-suite.org/tools/meme) was employed to assess conserved motifs in predicted alfalfa MsABC1Ks (Bailey et al., 2009); the obtained motifs were visualized using the TBtools software. The maximum pattern number determined was set as 10, with default values for the other parameters. Using the XinJiangDaYe genome annotation file, the MsABC1K gene structure was visualized using the TBtools software (v2.083). To identify *cis*-elements, the 2,000-bp promoter region located upstream of the ATG start codon of the alfalfa *MsABC1K* family members was uploaded to the PlantCARE website (http://bioinformatics.psb.ugent.be/webtools/plantcare/html/) (Lescot et al., 2002).

### 2.5 Secondary structure prediction, subcellular localization, and 3D structure prediction of alfalfa *MsABC1K* genes

BUSCA (http://busca.biocomp.unibo.it/) was utilized to predict the subcellular localization of the 22 *MsABC1K* genes. The secondary structure of the MsABC1K protein was predicted using the online tool SOPMA (http://npsa-pbil.ibcp.fr/cgi-bin/npsa_automat.pl?page=npsa_sopma.html). The online software ExPaSy SWISS-MODEL (https://swissmodel.expasy.org/interactive) was utilized to predict the 3D structure of the 22 MsABC1K proteins.

### 2.6 Planting of alfalfa plant materials and exposure to stress treatment

“Zhongmu No.1” alfalfa cultivar seeds were cultivated at the Institute of Grassland Research, Chinese Academy of Agricultural Sciences. The plants were cultured in a greenhouse at 25°C (day)/22°C (night) under a light/dark photoperiod (16/8 h). Samples of roots, leaves, stems, and flowers from a 2-month-old alfalfa plant were utilized to estimate tissue-specific expression. One-month-old alfalfa plants were treated with 300 mmol/L NaCl and 20% PEG-6000 to simulate salt stress and drought stress, respectively, and leaf samples were obtained at 0 h (CK) and 3, 6, and 12 h after treatment. Three biological replicates were used for each sample. The samples were placed in liquid nitrogen after sampling, frozen at −80°C, and then stored for quantitative reverse transcription–polymerase chain reaction (qRT−PCR) experiments.

### 2.7 RNA-seq analysis

The HiPure Polysaccharides & Polyphenolics Plant RNA Kit (Genepioneer, Beijing, China) was employed to extract total RNA. Sequencing libraries were generated using the NEBNext Ultra RNA Library Prep Kit for Illumina (NEB, Ipswich, MA, USA). The mRNA obtained from total RNA was purified with poly-T oligo-attached magnetic beads. cDNA was acquired by reverse transcription of the enriched mRNA. The purified double-stranded cDNA fragments were subjected to end repair, modified with A base addition, ligated to Illumina sequencing adapters, and sequenced using the Illumina NovaSeq X Plus system at the Nanjing Genepioneer Technology Company (Nanjing, China). The gene expression level was calculated based on the Fragments Per Kilobase of exon model per Million mapped fragments (FPKM) value. The TBtools software was utilized to visualize data.

### 2.8 RNA extraction and quantitative reverse transcription–polymerase chain reaction detection

The TransZol Up Plus RNA Kit (Transgene ER501, Beijing, China) was utilized to extract total RNA from different treatments. Reverse transcription was achieved using the HiScript II Q RT SuperMix for qPCR (+gDNA wiper) Kit (Vazyme R223, Nanjing, China). The Primer Premier 6 software was used to design specific primers (Singh et al., 1998). qRT-PCR amplification was conducted using the QuantStudio 5 PCR system (Thermo, Waltham, MA, USA). The *MsActin* gene was set as an internal control. qRT-PCR primers are shown in **Supplementary Table S1**. Experiments were performed with three replicates, and the 2^−ΔΔCt^ method was utilized to estimate the relative gene expression levels (Zhao et al., 2021; Wu et al., 2023). SPSS version 22.0 was used to perform statistical analyses. One-way ANOVA and Duncan’s test were used to determine differences in the parameters. Significant differences in mean values were assessed using Student’s t-test. * and ** indicate significant differences at *p* < 0.05 and 0.01, respectively.

## 3 Results

### 3.1 Alfalfa *MsABC1K* gene identification and characterization

Twenty-two *MsABC1K* genes in the alfalfa genome were recognized according to the HMM profile of the ABC1 domain (PF03109) sequence alignment and conserved structural domain analysis. The validated *ABC1K* genes were termed *MsABC1K1* to *MsABC1K15* based on their phylogenetic relationship with *AtABC1K*s. Allele genes located on the homologous chromosome were differentiated using lowercase letters a, b, and c. **Supplementary Table S2** shows the names, IDs, and amino acid sequences of the genes.

**Figure 1:**
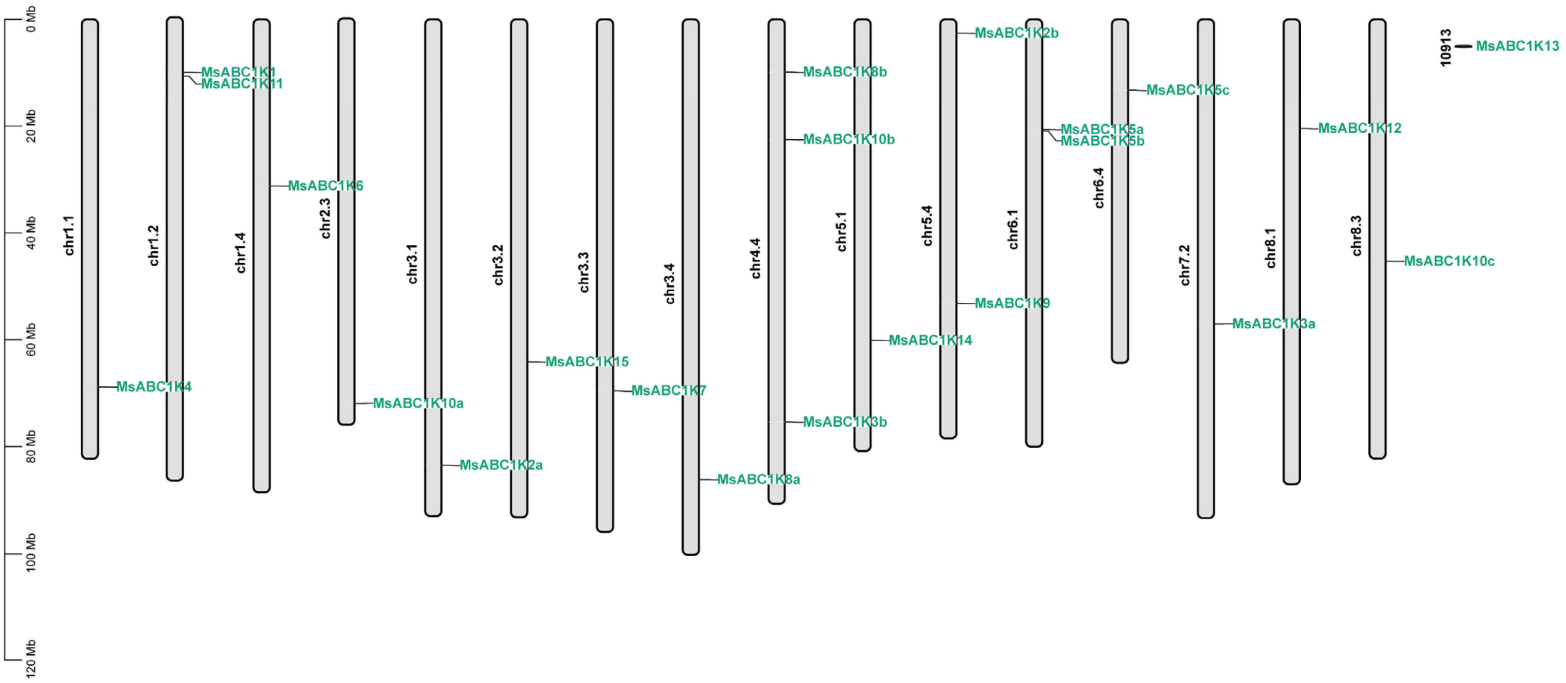
Chromosomal location of the identified *MsABC1K* genes on alfalfa chromosomes. The chromosomal localization of *MsABC1K* genes was determined based on the XinJiangDaYe genome annotation files.

The annotation data of the alfalfa genome showed that the 22 *MsABC1K* genes were distributed on 17 alfalfa chromosomes (**Figure 1**). Chr1.1, chr1.4, chr2.3, chr3.1, chr3.2, chr3.3, chr3.4, chr5.1, chr6.4, chr7.2, chr8.1, chr8.3, and mitochondrial chromosome 10913 contained only one *MsABC1K* gene. Three *MsABC1K* genes were present on chr4.4, and two *MsABC1K* genes were located on chr1.2, chr5.4, and chr6.1.

Physicochemical feature analysis showed that the coding sequence (CDS) of the alfalfa *ABC1K* genes was 963–2,877 bp in length and encoded 320–958 amino acids. The pI values and MW of the *MsABC1K* members were 5.29–9.58 and 36.26–106.13 kDa, respectively. The grand average of hydropathicity (GRAVY) values of the 22 MsABC1K proteins were negative, thus suggesting that the proteins were hydrophilic (**Supplementary Table S3**).

Among the 22 MsABC1Ks, the subcellular localization prediction showed that MsABC1K1, MsABC1K2a, MsABC1K5b, MsABC1K9, and MsABC1K13 were located in chloroplasts and that MsABC1K2b/3a/5a/6/7/8b/10b/11/12/14/15 were located in the organelle membrane and endomembrane system. MsABC1K3b/5c/8a/10a/10c and MsABC1K4 were localized in the mitochondria and nucleus, respectively (**Supplementary Table S3**).

The 3D structures of all 22 MsABC1K proteins were predicted using ExPaSy SWISS-MODEL (**Supplementary Figure S1**). The MsABC1K proteins were mainly constructed based on α-helices and random coils, and most of the MsABC1K proteins had more than 50% distribution of α-helices (**Supplementary Table S4**).

### 3.2 Phylogenetic analysis results and grouping of MsABC1Ks in alfalfa

**Figure 2:**
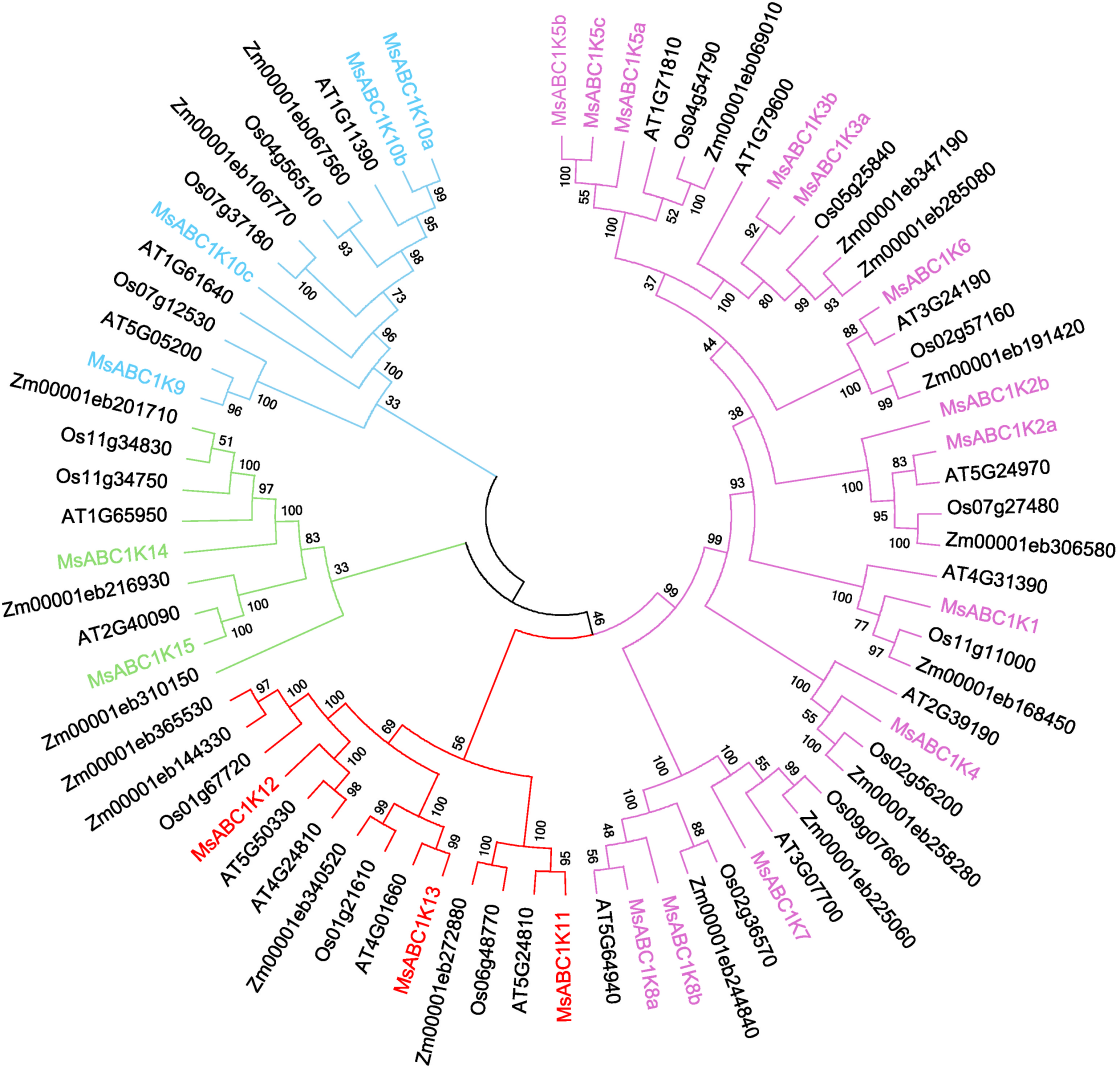
Phylogenetic tree of *MsABC1K* genes from *Medicago sativa*, *Arabidopsis thaliana*, *Oryza sativa*, and *Zea mays*. The phylogenetic tree was constructed using ABC1K amino acid sequences by the neighbor-joining (NJ) method with 1,000 bootstrap replicates. The phylogenetic tree was divided into four groups shown in different colors.

To determine the evolutionary relationship of MsABC1K proteins with ABC1K proteins of other species, the complete protein sequences of 22 MsABC1Ks, 17 AtABC1Ks, 16 OsABC1Ks, and 19 ZmABC1Ks were utilized to construct an unrooted phylogenetic tree (**Supplementary Table S5**). The 22 MsABC1K members were classified into four groups (**Figure 2**). Among these groups, subfamily I had the most members, including MsABC1K1/2a/2b/3a/3b/4/5a/5b/5c/6/7/8a/8b. Subfamily II had four members, including MsABC1K9/10a/10b/10c. Subfamily III had three members, namely, MsABC1K11/12/13. Subfamily IV had two members, MsABC1K14 and MsABC1K15.

### 3.3 Determination of *cis*-regulatory elements, gene structure, and conserved motifs of MsABC1Ks

**Figure 3:**
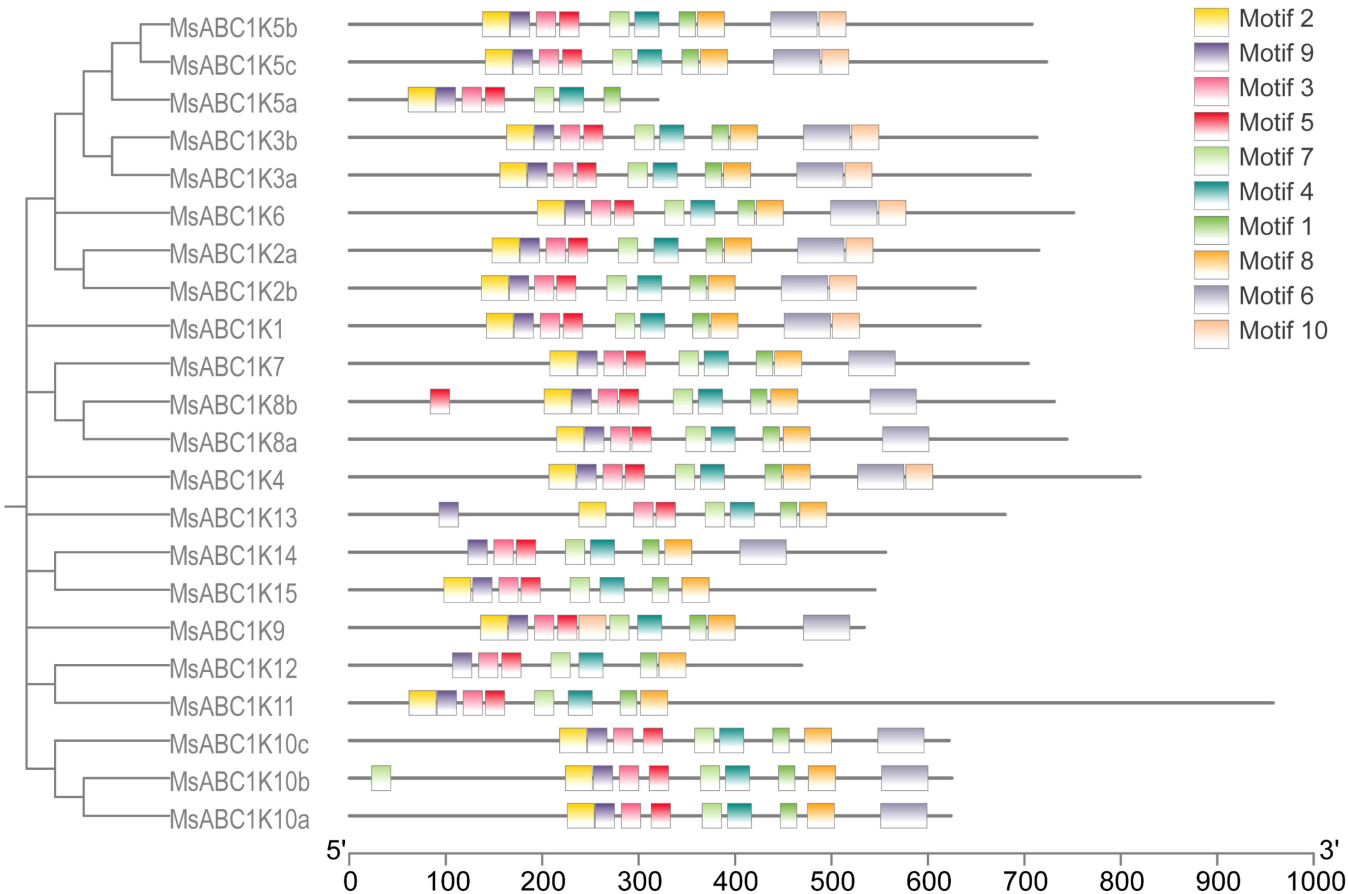
Analysis of conserved motifs in *MsABC1K* genes. Ten types of conserved motifs were predicted in the MsABC1K protein sequences. The different motifs are shown in different color boxes.

To discover the conserved motifs of MsABC1Ks, the MEME online tool was utilized to analyze the motif distribution of all 22 MsABC1Ks, and 10 conserved motifs were detected (**Figure 3**). Motifs 1, 3, 4, 5, 7, and 9 exhibited high conservation across all MsABC1Ks. Motif 2 was not detected in MsABC1K12 or MsABC1K14. Motif 6 was not found in the subfamily I member MsABC1K5a; the subfamily III members MsABC1K11, MsABC1K12, and MsABC1K13; or the subfamily IV member MsABC1K15. Motif 8 was not found in the subfamily I member MsABC1K5a. Motif 10 was not detected in the subfamily I members MsABC1K5a, MsABC1K7, MsABC1K8a, or MsABC1K8b.

**Figure 4:**
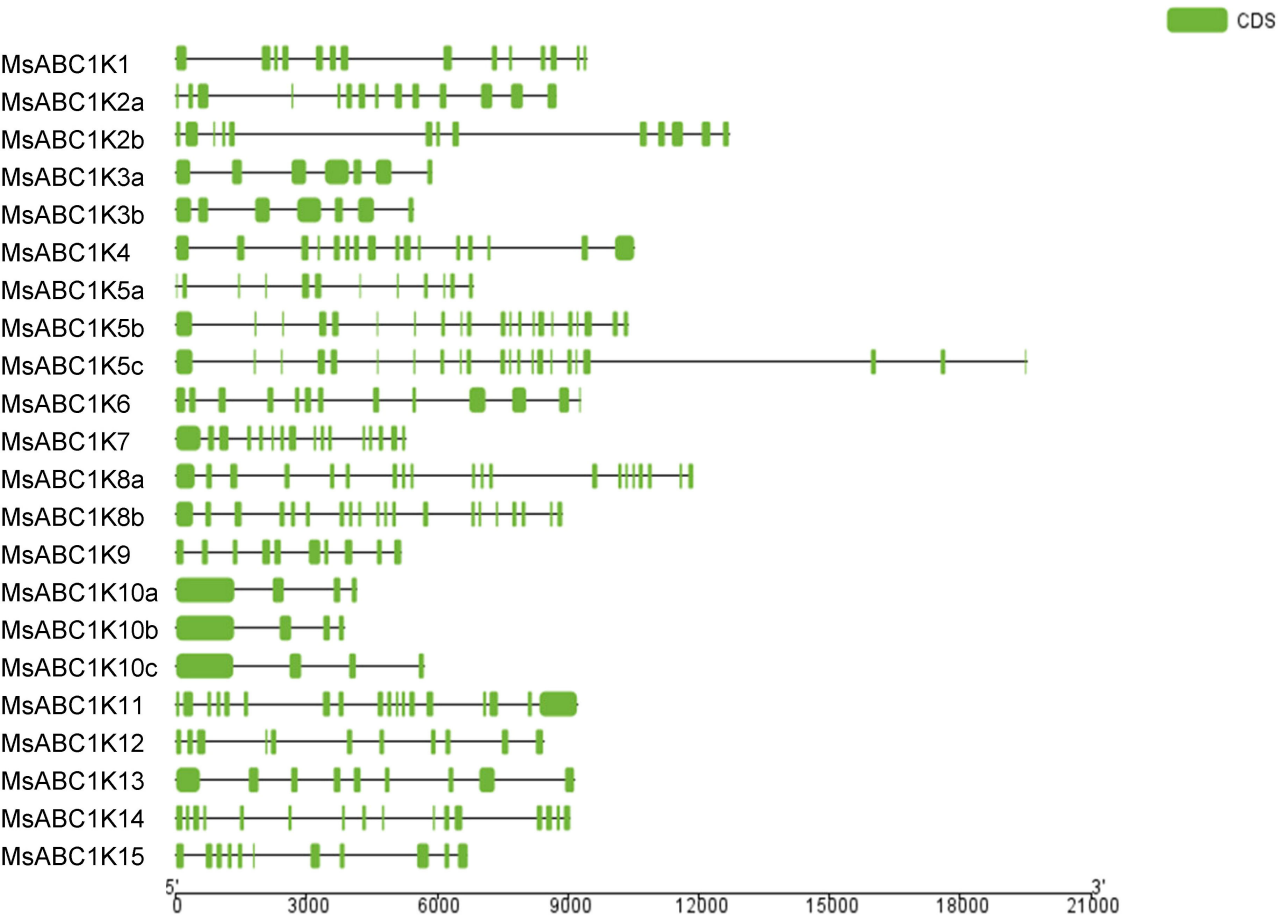
The gene structure of *MsABC1K* members. (Left) Phylogenetic analysis of the *MsABC1K* gene family constructed using MEGA 7.0 software with the neighbor-joining (NJ) method; (right) gene structures of the 22 *MsABC1K* genes were analyzed. Exons and introns are shown as green boxes and horizontal lines, respectively.

To determine the *MsABC1K* gene structural composition, we analyzed the intron–exon structure of MsABC1Ks. *MsABC1K* gene structure showed differences as well as similarities among the four subfamilies (**Figure 4**). Most of the genes (18 of 22, 81.82%) had seven exons or more, and only three genes, namely, *MsABC1K10a*, *MsABC1K10b*, and *MsABC1K10c*, had four exons. In subfamily I, the number of exons was 7–22, which widely fluctuated as compared to that in the other subfamilies. In subfamily II, *MsABC1K9* had 10 exons, which varied from that noted in *MsABC1K10a*/*MsABC1K10b*/*MsABC1K10c*. Subfamily III members had 9–18 exons, while subfamily IV members had 11 or 16 exons.

**Figure 5:**
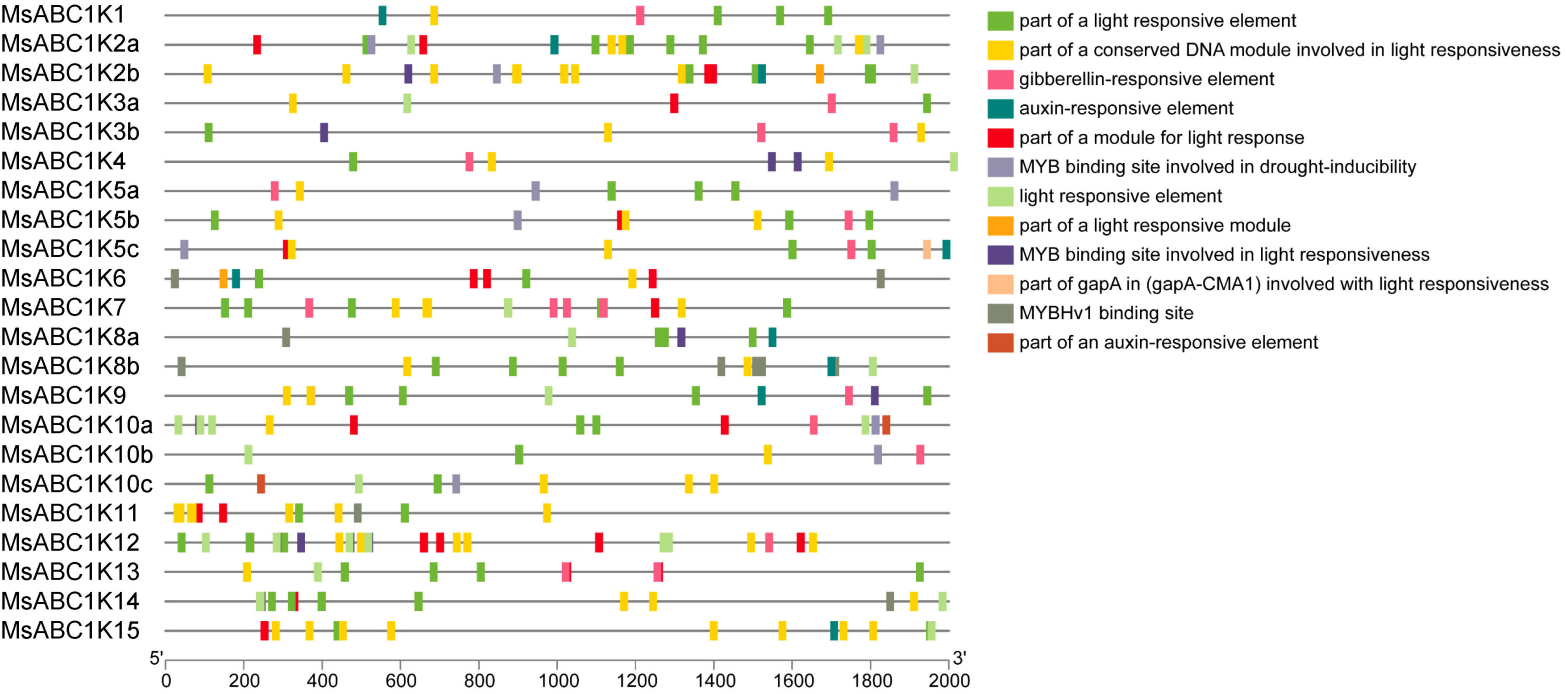
Prediction of *cis*-acting elements in the promoter regions of *MsABC1K*s. (Left) Distribution of *cis*-elements in the promoter sequences of *MsABC1K*s. (Right) The boxes with different colors represent different *cis*-elements.

By employing a 2.0-kb promoter sequence located upstream of the *MsABC1K* gene start codon, we discovered the *cis*-elements are potentially involved in the transcriptional regulation of *MsABC1K*. We speculated that most *cis*-regulatory elements showed an association with light responsiveness in the alfalfa *MsABC1K* gene promoter region, some of which were specifically related to hormone and drought responsiveness (**Figure 5**). Thus, *MsABC1K* gene expression is potentially controlled by diverse light-responsive regulatory elements, phytohormones, and abiotic stresses. Different *MsABC1K* genes had similar *cis*-acting elements, which reflected the evolutionary conservation of the *MsABC1K* gene family.

### 3.4 Determination of *MsABC1K* expression in various tissues

**Figure 6:**
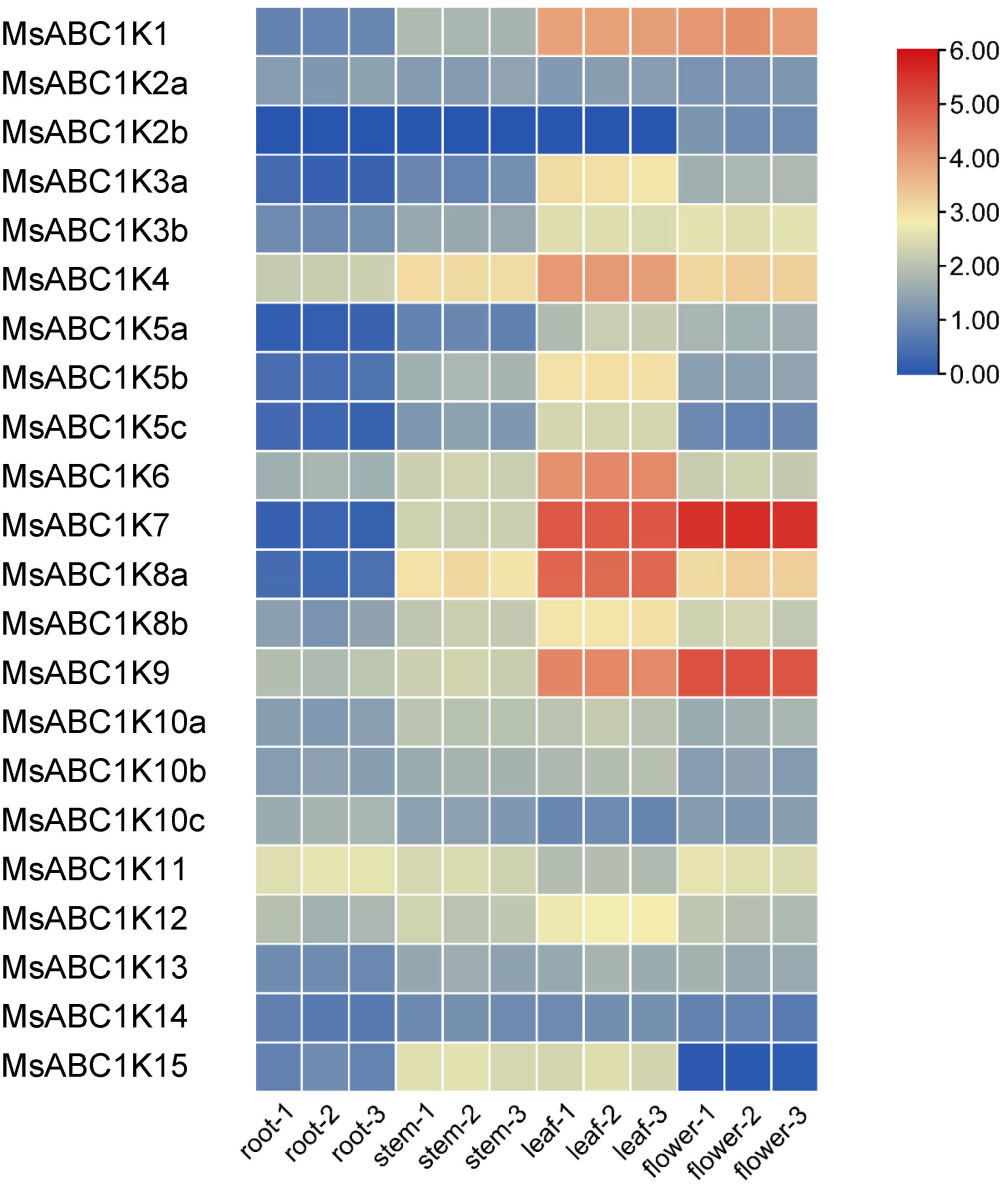
Expression levels of *MsABC1K*s in different tissues. Transcriptome sequencing was used to analyze the samples of roots, stems, leaves, and flowers collected from 2-month-old alfalfa plants. The number represents three independent biological replicates. The gene expression level was calculated based on the FPKM value. Red and blue represent high and low expression levels, respectively. The raw data are provided in **Supplementary Table S6**.

To clarify the *MsABC1K* expression pattern in different alfalfa tissues, transcriptome sequencing was performed to analyze *MsABC1K* gene expression in alfalfa stems, roots, leaves, and flowers (**Figure 6**). Twenty-one *MsABC1K* genes were expressed in all four alfalfa tissues; however, *MsABC1K2b* was expressed only in flowers. *MsABC1K* gene expression abundance significantly varied among the different tissues. The *MsABC1K* genes *MsABC1K1/3a/3b/4/5a/6/7/8a/8b/9* exhibited preferential expression in leaves and flowers, while the *MsABC1K* genes *MsABC1K5b/5c/12/15* exhibited preferential expression in stems and leaves. Additionally, the *MsABC1K* genes *MsABC1K2a/10a/10b/10c/11/13/14* showed similar expression patterns in different tissues. These results demonstrate that *MsABC1K* genes perform distinct functions during normal development and growth.

### 3.5 Assessment of *MsABC1K* gene expression following salt stress exposure

**Figure 7:**
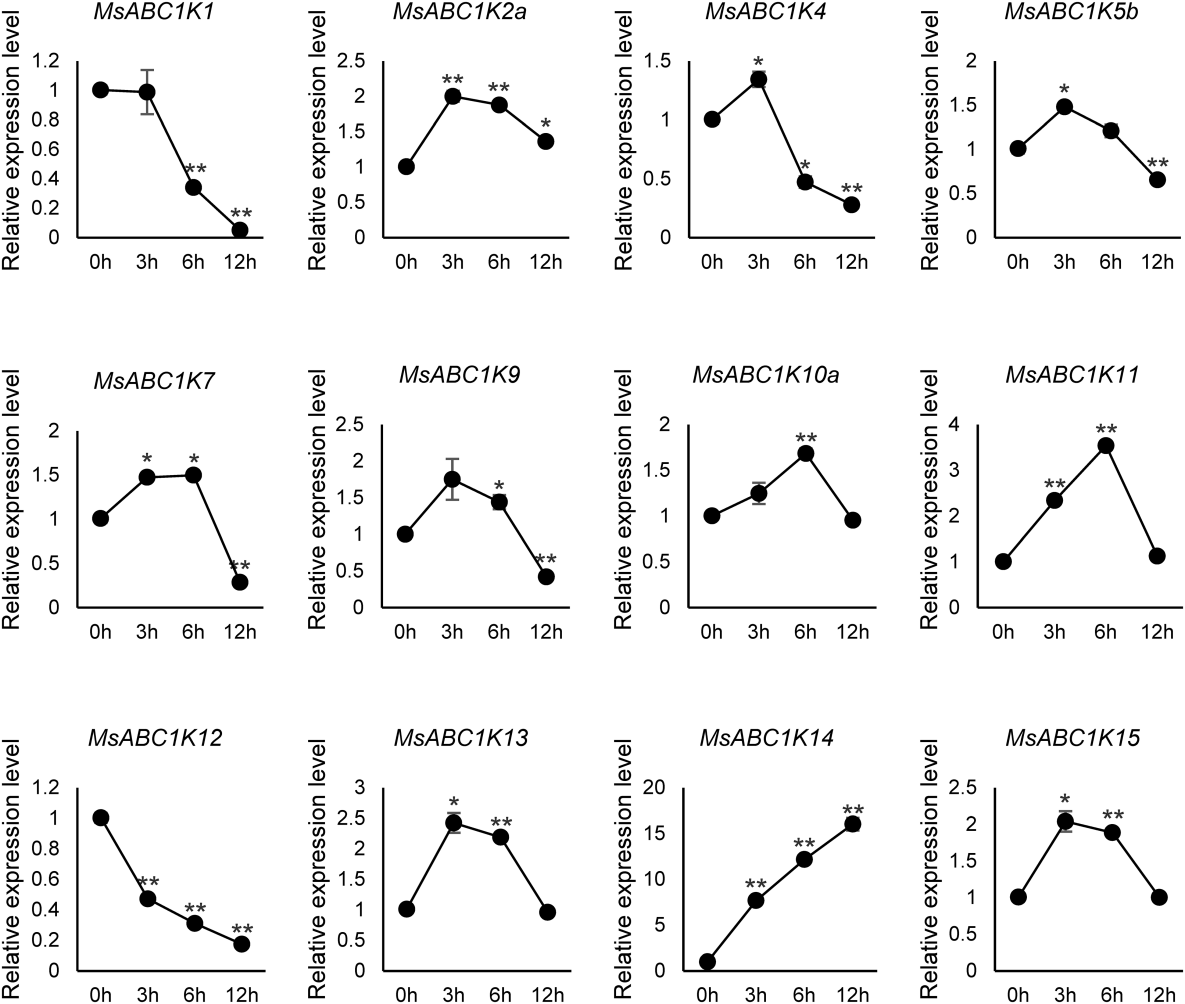
Expression analysis of 12 *MsABC1K*s after NaCl treatment. qRT-PCR analysis of the mRNA expression levels of *MsABC1K*s in the leaves of alfalfa plants at 0 h, 3 h, 6 h, and 12 h after 300 mM NaCl treatment. The *MsActin* gene was used as the internal reference gene. Asterisks indicate significant differences (Student’s t-test, *p < 0.05, **p < 0.01).

To examine whether *MsABC1K* genes participate in salt stress response in alfalfa, we conducted qRT-PCR to detect the leaf expression profiles of 12 *MsABC1K*s belonging to four different subfamilies following treatment with 300 mM NaCl for various time periods: 0 h, 3 h, 6 h, and 12 h (**Figure 7**). Six *MsABC1K* genes (*MsABC1K2a/10a/11/13/14/15*) showed a significant increase in their expression levels; among these genes, the expression level of *MsABC1K14* was 16-fold at 12 h after NaCl treatment. Four *MsABC1K* genes (*MsABC1K4*, *MsABC1K5b*, *MsABC1K7*, and *MsABC1K9*) exhibited significantly increased expression in the early stage, which finally decreased; in contrast, *MsABC1K1* and *MsABC1K12* gene expression was remarkably suppressed after salt stress exposure. Moreover, following treatment with 300 mM NaCl, 12 *MsABC1K* genes displayed relatively complicated expression patterns at 3 h, 6 h, and 12 h, possibly indicating a different regulatory function of each *MsABC1K* gene in alfalfa after exposure to salt stress.

### 3.6 Evaluation of *MsABC1K* gene expression following drought stress exposure

**Figure 8:**
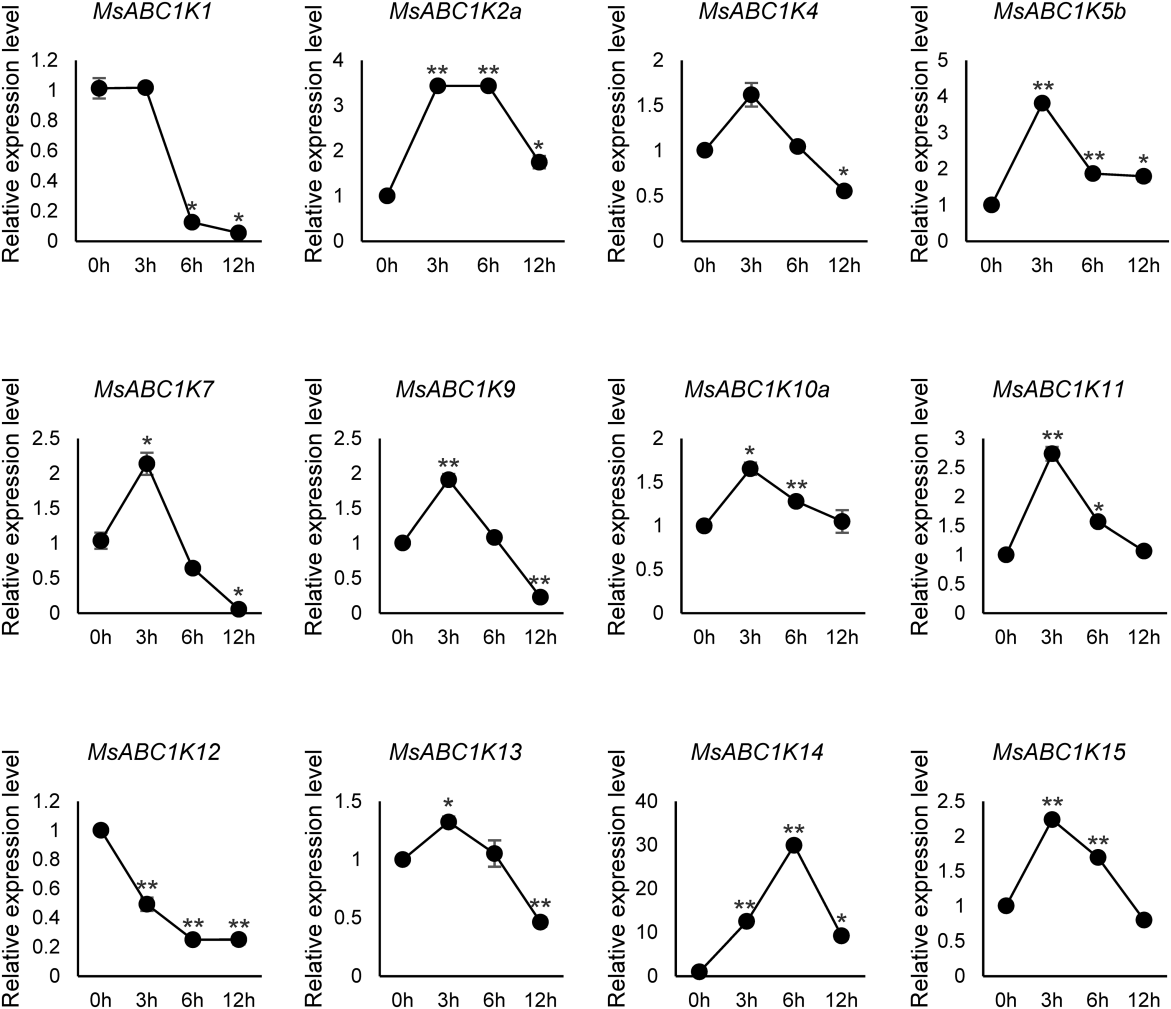
Expression analysis of 12 *MsABC1K* genes after PEG-6000 treatment. qRT-PCR analysis of the mRNA expression levels of *MsABC1K*s in the leaves of alfalfa plants at 0 h, 3 h, 6 h, and 12 h after 20% PEG-6000 treatment. The *MsActin* gene was used as the internal reference gene. Asterisks indicate significant differences (Student’s t-test, *p < 0.05, **p < 0.01).

To better comprehend the response of *MsABC1K* genes under drought stress, we conducted qRT-PCR analysis of alfalfa following treatment with 20% (w/v) PEG-6000. As shown in **Figure 8**, 12 *MsABC1K* genes responded to drought stress. These genes were clustered into two groups according to expression patterns: group I included two genes (*MsABC1K1* and *MsABC1K12*) with significant downregulation in drought stress, and group II included 10 *MsABC1K* genes with upregulated expression in drought stress. The 10 *MsABC1K* members in the second group showed peak expression levels at varying time points following PEG-6000 treatment. In this case, the expression abundance of the *MsABC1K* genes *MsABC1K2a/4/5b/7/9/10a/11/13/15* peaked at 3 h following drought stress exposure, while *MsABC1K14* expression abundance peaked at 6 h following drought stress treatment. These findings confirmed that *MsABC1K* genes are generally involved and have critical functions in alfalfa’s response to abiotic stress.

## 4 Discussion

The ABC1K atypical protein kinase families widely occur in several plants, and their number varies among different species (Gao et al., 2011; Jasinski et al., 2008; Yang et al., 2012). The current study identified 22 *MsABC1K* genes in the alfalfa (XinJiangDaYe cultivar) genome. We conducted primary bioinformatics analysis, sequence determination, evolution analysis, and expression analysis. Based on the obtained results, the *MsABC1K* genes may perform important functions in alfalfa development, growth, and resistance to various abiotic stress conditions such as salinity and drought.

Phylogenetic tree analysis classified the 22 *MsABC1K* genes into four subfamilies according to their structural domains (**Figure 2**), which was different from that of *Arabidopsis*, rice, and maize (Lundquist et al., 2012a). There were 13, 4, 3, and 2 *MsABC1K* genes in subfamilies I, II, III, and IV, respectively. The *MsABC1K* genes were appropriately allocated into the known groups of *Arabidopsis thaliana*, rice, and maize; these findings revealed that alfalfa *MsABC1K* genes were evolutionarily conserved. Gene structure analysis demonstrated similar exon/intron structure for the genes in the same subfamily (**Figure 4**). Furthermore, according to conserved motif analysis, the genes in the same subfamily generally included the conserved motifs (**Figure 3**), which indicated that these MsABC1K proteins possibly show identical functions.

Transcriptional regulation, a tedious and complex biological process, directly affects gene expression levels and patterns and influences stress resistance/tolerance, phenotype, and plant productivity (Cramer, 2019; Cui et al., 2023). To predict the pathways in which MsABC1Ks participate, *cis*-elements in the promoters of the *MsABC1K* genes were assessed (**Figure 5**). All 22 *MsABC1K* genes had light-responsive elements, thus indicating that light participated in regulating *MsABC1K* gene expression. Gibberellin and auxin, plant phytohormones, are involved in plant developmental regulation and stress responsiveness in some plant species (Bao et al., 2020; Cavallari et al., 2021; Gomes and Scortecci, 2021; Kunkel and Johnson, 2021; Liu J. et al., 2023; Wang and Wang, 2022). In the present study, 13 of 22 *MsABC1K* genes (*MsABC1K1/3a/3b/4/5a/5b/5c/7/9/10a/10b/12/13*) contained the gibberellin-responsive element, while 10 of 22 *MsABC1K* genes (*MsABC1K1/2a/2b/5c/6/8a/8b/9/10c/15*) contained the auxin-responsive element; this finding suggested that gibberellin and auxin can alter the expression level of the *MsABC1K* genes and subsequently affect the growth and development of plants. We also observed that eight *MsABC1K* genes (*MsABC1K2a/2b/5a/5b/5c/10a/10b/10c*) had the drought-inducibility element, which suggested that these *MsABC1K* genes are probably drought-regulated and respond to other drought regulation-related genes. Taken together, our results clarified that *MsABC1K* genes may perform critical functions in the growth and developmental pathways as well as in the abiotic stress responsiveness of alfalfa.

According to previous studies, the ABC1K protein family participates in monitoring processes related to plant development and growth and stress adaptation (Manara et al., 2015, 2016; Yang et al., 2016; Qin et al., 2020). Although ABC1K homologs have crucial functions in other species, little information is currently available regarding ABC1K homologs’ role in alfalfa. We examined tissue-specific expression patterns of MsABC1K proteins and found unique expression patterns of different MsABC1K proteins in the four tissues (**Figure 6**). Our results showed that most MsABC1K proteins had relatively high expression levels in leaves and flowers, which implied their corresponding *MsABC1K* genes may contribute to leaf and flower development.

Abiotic stress, one of the primary environmental stress factors, limits the development, growth, and value of alfalfa (Guo et al., 2020; Guo et al., 2024; Mi et al., 2024; Zhou et al., 2024). Here, *MsABC1K* gene expression levels were quantified in leaves after NaCl (300 mM) and PEG-6000 (20% [w/v]) treatments to elucidate whether *MsABC1K* genes respond to salt stress and drought stress, respectively. Ten *MsABC1K* genes (10 of 12, 83.3%) were upregulated following NaCl treatment, while nine (9 of 12, 75%) *MsABC1K* genes were upregulated following PEG-6000 treatment; most of these genes showed peak expression levels at different time points (**Figures 7**, **8**). The expression levels of *MsABC1K2a*, *MsABC1K5b*, and *MsABC1K10a* genes were upregulated, which was consistent with the prediction results of *cis*-elements, thus indicating that *MsABC1K2a*, *MsABC1K5b*, and *MsABC1K10a* play important roles in response to drought stress. The *MsABC1K2a/5b/7/9/10a/11/13/14/15* genes showed similar expression patterns under these two types of stresses, thus indicating that these *MsABC1K* genes functioned as positive regulatory factors in alfalfa for defense against different abiotic stresses. *MsABC1K14* was significantly upregulated by more than 10-fold; thus, it can be used as an important target gene for further analysis of the function of *MsABC1K*s. The *Arabidopsis* genes *ABC1K7* and *ABC1K8* mediate the crosstalk of ABA with ROS signaling (Manara et al., 2016), and ABA influences abiotic stress response as well as plant development (Verma et al., 2016; Wei et al., 2022; Yang et al., 2022). Our study revealed a remarkable increase in the *MsABC1K7* gene expression level within a short time after exposure to salt and drought stresses, thus indicating the positive regulatory role of this gene in different abiotic stress conditions through the ABA signaling pathway; this assumption needs to be further confirmed. We identified 22 *MsABC1K* genes from the tetraploid alfalfa genome and noted the activation of many *MsABC1K* members following salt and drought stress exposure. Future research should focus on further functional verification of these genes to better comprehend the key roles and mechanisms of *MsABC1K* genes under abiotic stress.

## 5 Conclusions

We performed genome-wide identification of 22 putative *MsABC1K* genes located on different chromosomes from the tetraploid alfalfa genome. Phylogenetic tree analysis categorized *MsABC1K* genes into four subfamilies; this finding varied from the results for *Arabidopsis*. The expression pattern evaluation of these *MsABC1K* genes in four different alfalfa tissues revealed that 21 *MsABC1K* genes were expressed in all four tissues studied, except for *MsABC1K2b*, and 14 of the 22 *MsABC1K* genes showed tissue-specific expression. qRT-PCR analysis demonstrated that 10 of 12 *MsABC1K* genes were upregulated following NaCl treatment, and nine of 12 *MsABC1K* genes were upregulated after PEG-6000 treatment. The expression of both *MsABC1K1* and *MsABC1K12* was remarkably suppressed under salt and drought stresses. Thus, our study provides novel data to more comprehensively examine functions performed by the *MsABC1K* gene family; further research is required to develop approaches to improve alfalfa plant quality after exposure to salt and drought stresses.

## Data Availability

The datasets presented in this study can be found in online repositories. The names of the repository/repositories and accession number(s) can be found in the article/**Supplementary Material**.

## Supplementary material

Supplementary Figure 1 Three-dimensional structures of 22 MsABC1K proteins predicted by ExPaSy SWISS-MODEL.
