# Supplementary figures and images for: Genome-wide identification and analysis of expression patterns of the ABC1K gene family members in Medicago sativa
Source: Front Plant Sci. 2024 Nov 25;15:1486525. doi: 10.3389/fpls.2024.1486525 (PMC11625579; doi:10.3389/fpls.2024.1486525)

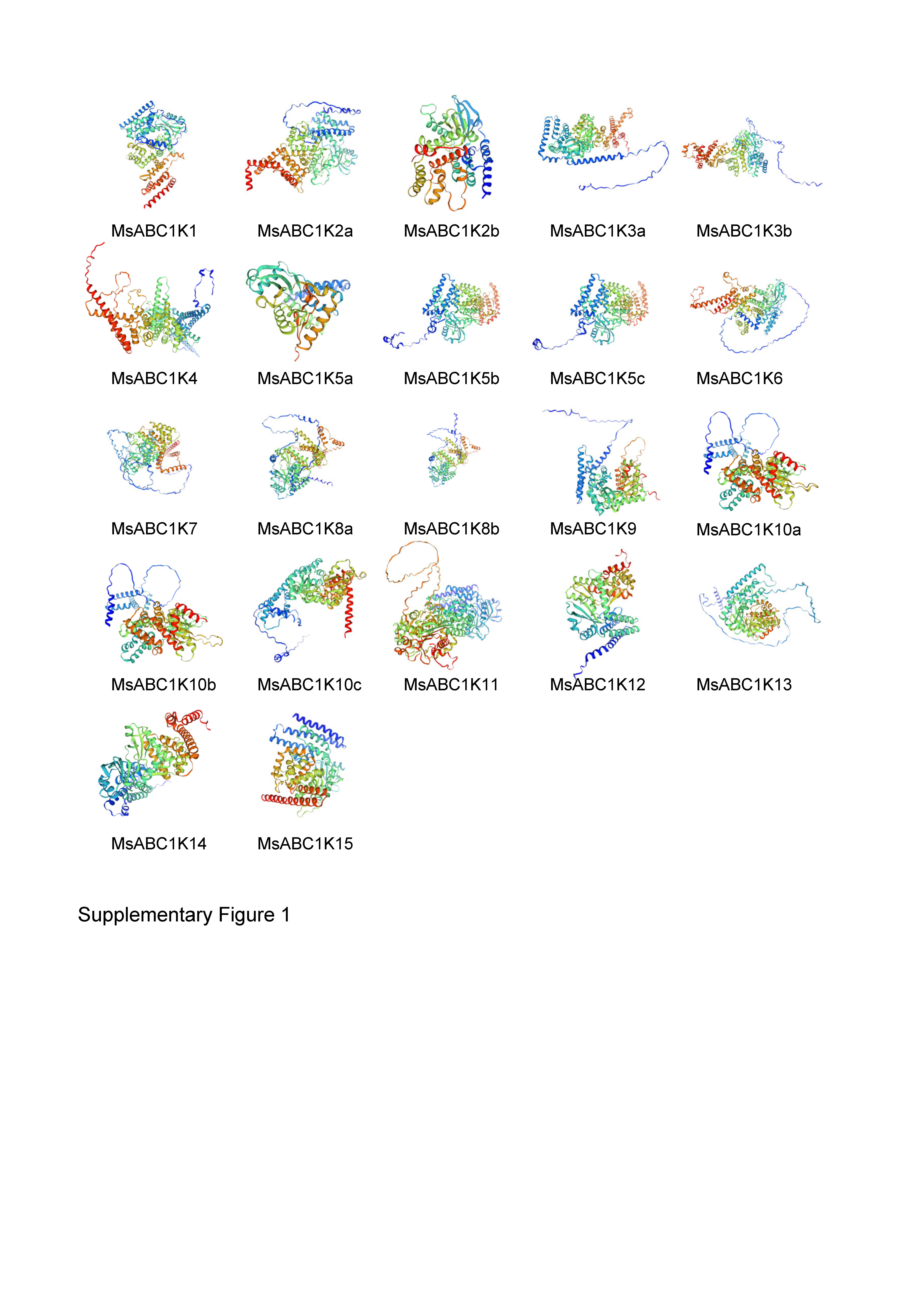

Supplement: Supplementary Figure 1 — Three-dimensional structures of 22 MsABC1K proteins predicted by ExPaSy SWISS-MODEL. [file Image1.tif]
