# Supplementary material for: Genome-wide identification and analysis of expression patterns of the ABC1K gene family members in Medicago sativa
Source: Front Plant Sci. 2024 Nov 25;15:1486525. doi: 10.3389/fpls.2024.1486525 (PMC11625579; doi:10.3389/fpls.2024.1486525)
Supplement: Supplementary file 2 [file Table1.docx]

**Supplementary Table 1. Primers used in qRT-PCR.**

| Primer name | Primer sequence（5’-3’） |
| --- | --- |
| MsABC1K1-F | TATTGGTCTGCCTTGGT |
| MsABC1K1-R | GATGAGATGCTGCTGAAA |
| MsABC1K2a-F | CCGAAGACCCTAATCCT |
| MsABC1K2a-R | CCAACTTCACCACCTCAC |
| MsABC1K4-F | GTCATCCGCCAACAACT |
| MsABC1K4-R | ATTAGCCAACATCACCCT |
| MsABC1K5b-F | ACGGCAAAGTCAGGGTA |
| MsABC1K5b-R | CAAATCGGAACGAGACG |
| MsABC1K7-F | CCTCTGGTTTATTGGGATT |
| MsABC1K7-R | ATGGCTGGTCTTGTGCT |
| MsABC1K9-F | ATGGCGGTTTCAGGGTT |
| MsABC1K9-R | ACGCAGCGGATGAGTTT |
| MsABC1K10a-F | TCCCTAAGCCTGTCTATC |
| MsABC1K10a-R | TTACCCTATCACTACCAGAG |
| MsABC1K11-F | ATTTGGCTGGTTGTGCG |
| MsABC1K11-R | GCTGTGGCTGGAAGGTA |
| MsABC1K12-F | GTTCAGTTTCGTCCTATTCA |
| MsABC1K12-R | GAGCGTAACCAGCCTTT |
| MsABC1K13-F | CTACTTCGCAAGGCAATC |
| MsABC1K13-R | GCAGCAGCTAAAGGTTCA |
| MsABC1K14-F | GGATGTCTCACGGCTCA |
| MsABC1K14-R | GAAGAAAGGCGGAATGG |
| MsABC1K15-F | TGGAGCGACACCAGATA |
| MsABC1K15-R | GTTGGACCTTCACAGCA |
| MsActin-F | CAAAAGATGGCAGATGCTGAGGAT |
| MsActin-R | CATGCACCAGTATGACGAGGTCG |
