# Supplementary material for: Genome-wide identification and analysis of expression patterns of the ABC1K gene family members in Medicago sativa
Source: Front Plant Sci. 2024 Nov 25;15:1486525. doi: 10.3389/fpls.2024.1486525 (PMC11625579; doi:10.3389/fpls.2024.1486525)
Supplement: Supplementary file 4 [file Table3.docx]

**Supplementary Table 3.** **Nomenclature and characteristics of MsABC1K family genes in *M. Sativa*.**

| Gene name | Gene ID | PI | Mw(kDa) | CDS length | Protein length | Locations on chromosome | GRAVY | Subcellular localization |
| --- | --- | --- | --- | --- | --- | --- | --- | --- |
| MsABC1K1 | MS.gene54068 | 5.29 | 73.38 | 2043 | 654 | 1B: 10,323,658-10,333,078 | -0.024 | chloroplast |
| MsABC1K2a | MS.gene014926 | 8.24 | 81.24 | 2148 | 715 | 3A: 83,466,148-83474,887 | -0.105 | chloroplast |
| MsABC1K2b | MS.gene024816 | 9.08 | 73.38 | 1950 | 649 | 5D: 2,581,657-2,594,351 | -0.033 | organelle membrane |
| MsABC1K3a | MS.gene20121 | 5.91 | 78.79 | 2121 | 706 | 7B: 57,043,790-57,049,676 | -0.021 | organelle membrane |
| MsABC1K3b | MS.gene059247 | 6.22 | 79.87 | 2142 | 713 | 4D: 75,406,411-75,411,865 | -0.059 | Mitochondria |
| MsABC1K4 | MS.gene041784 | 9.58 | 92.03 | 2463 | 820 | 1A: 68,825,619-68,836,140 | -0.076 | nucleus |
| MsABC1K5a | MS.gene52872 | 9.17 | 36.26 | 963 | 320 | 6A: 20,666,429-20,673,246 | -0.074 | organelle membrane |
| MsABC1K5b | MS.gene52883 | 6.28 | 79.11 | 2127 | 708 | 6A: 20,898,605-20,908,985 | -0.001 | chloroplast |
| MsABC1K5c | MS.gene69300 | 5.55 | 81.21 | 2172 | 723 | 6D: 13,263,844-13,283,370 | -0.097 | Mitochondria |
| MsABC1K6 | MS.gene064971 | 5.43 | 82.86 | 2257 | 751 | 1D: 31,211,498-31,220,789 | -0.045 | organelle membrane |
| MsABC1K7 | MS.gene000091 | 9.33 | 79.95 | 2115 | 704 | 3C: 69,542,709-69,547,972 | -0.310 | organelle membrane |
| MsABC1K8a | MS.gene013394 | 8.88 | 83.76 | 2235 | 744 | 3D: 86,177,493-86,189,369 | -0.247 | Mitochondria |
| MsABC1K8b | MS.gene28132 | 9.04 | 82.66 | 2196 | 731 | 4D: 9,882,049-9,890,918 | -0.231 | organelle membrane |
| MsABC1K9 | MS.gene023767 | 7.10 | 59.79 | 1605 | 534 | 5D: 53,204,066-53,209,239 | -0.007 | chloroplast |
| MsABC1K10a | MS.gene002356 | 9.50 | 71.14 | 1875 | 624 | 2C: 72,116,032-72,120,183 | -0.182 | Mitochondria |
| MsABC1K10b | MS.gene32221 | 9.33 | 70.47 | 1878 | 625 | 4D: 22,505,280-22,509,135 | -0.078 | organelle membrane |
| MsABC1K10c | MS.gene046482 | 9.26 | 70.80 | 1869 | 622 | 8C: 45,332,401-45,338,093 | -0.054 | Mitochondria |
| MsABC1K11 | MS.gene059105 | 7.12 | 106.13 | 2877 | 958 | 1B: 11,000,147-11,009,354 | -0.235 | endomembrane system |
| MsABC1K12 | MS.gene48278 | 8.09 | 52.84 | 1410 | 469 | 8A: 20,425,914-20,434,341 | -0.084 | endomembrane system |
| MsABC1K13 | MS.gene21118 | 5.74 | 74.20 | 2043 | 680 | 10913: 69496-78636 | -0.198 | chloroplast |
| MsABC1K14 | MS.gene017355 | 9.22 | 63.40 | 1671 | 556 | 5A: 60,100,629-60,109,679 | -0.072 | organelle membrane |
| MsABC1K15 | MS.gene69233 | 8.47 | 61.71 | 1638 | 545 | 3B: 64,147,621-64,154,317 | -0.127 | endomembrane system |

**Supplementary Table 4.** **Percentage of α-helix, extended strand and random coil of MsABC1K proteins.**

| **Gene name** | **Alpha helix (Hh)** | **Extended strand (Ee)** | **Random coil (Cc)** |
| --- | --- | --- | --- |
| MsABC1K1 | 343(52.45%) | 55 (8.41%) | 256 (39.14%) |
| MsABC1K2a | 401(56.08%) | 39(5.45%) | 275 (38.46%) |
| MsABC1K2b | 395(60.86%) | 43 (6.63%) | 211 (32.51%) |
| MsABC1K3a | 412 (58.36%) | 47 (6.66%) | 247 (34.99%) |
| MsABC1K3b | 405 (56.80%) | 43 (6.03%) | 265 (37.17%) |
| MsABC1K4 | 465 (56.71%) | 60 (7.32%) | 295 (35.98%) |
| MsABC1K5a | 169 (52.81%) | 43 (13.44%) | 108 (33.75%) |
| MsABC1K5b | 405 (57.20%) | 53 (7.49%) | 250 (35.31%) |
| MsABC1K5c | 375 (51.87%) | 53 (7.33%) | 295 (40.80%) |
| MsABC1K6 | 375 (49.93%) | 51 (6.79%) | 325(43.28%) |
| MsABC1K7 | 341(48.44%) | 72(10.23%) | 291(41.34%) |
| MsABC1K8a | 377(50.67%) | 70(9.41%) | 297(39.92%) |
| MsABC1K8b | 444(60.74%) | 55(7.52%) | 232(31.74%) |
| MsABC1K9 | 302(56.55%) | 34(6.37%) | 198(37.08%) |
| MsABC1K10a | 301(48.24%) | 80(12.82%) | 243(38.94%) |
| MsABC1K10b | 339(54.24%) | 57(9.12%) | 229(36.64%) |
| MsABC1K10c | 316(50.80%) | 75(12.06%) | 231(37.14%) |
| MsABC1K11 | 402(41.96%) | 83(8.66%) | 473(49.37%) |
| MsABC1K12 | 276 (58.85%) | 32(6.82%) | 161(34.33%) |
| MsABC1K13 | 296 (43.53%) | 62(9.12%) | 322(47.35%) |
| MsABC1K14 | 337 (60.61%) | 45(8.09%) | 174(31.29%) |
| MsABC1K15 | 272 (49.91%) | 68(12.48%) | 205(37.61%) |

**Supplementary Table 5. Gene IDs of the ABC1K gene family members from four plant species.**

| **Spicies** | **Gene ID** | **Spicies** | **Gene ID** | **Spicies** | **Gene ID** |
| --- | --- | --- | --- | --- | --- |
| Medicago sativa | MS.gene54068 | Arabidopsis thaliana | AT2G39190 | Oryza sativa | Os06g48770 |
|  | MS.gene014926 |  | AT1G71810k |  | Os01g67720 |
|  | MS.gene024816 |  | AT3G24190 |  | Os01g21610 |
|  | MS.gene20121 |  | AT3G07700 |  | Os11g34750 |
|  | MS.gene059247 |  | AT5G64940 |  | Os11g34830 |
|  | MS.gene041784 |  | AT5G05200 | Zea mays | Zm00001eb168450 |
|  | MS.gene52872 |  | AT1G11390 |  | Zm00001eb306580 |
|  | MS.gene52883 |  | AT1G61640 |  | Zm00001eb347190 |
|  | MS.gene69300 |  | AT5G24810 |  | Zm00001eb285080 |
|  | MS.gene064971 |  | AT4G24810 |  | Zm00001eb258280 |
|  | MS.gene000091 |  | AT5G50330 |  | Zm00001eb069010 |
|  | MS.gene013394 |  | AT4G01660 |  | Zm00001eb191420 |
|  | MS.gene28132 |  | AT1G65950 |  | Zm00001eb225060 |
|  | MS.gene023767 |  | AT2G40090 |  | Zm00001eb244840 |
|  | MS.gene002356 | Oryza sativa | Os11g11000 |  | Zm00001eb303700 |
|  | MS.gene32221 |  | Os07g27480 |  | Zm00001eb106770 |
|  | MS.gene046482 |  | Os05g25840 |  | Zm00001eb067560 |
|  | MS.gene059105 |  | Os02g56200 |  | Zm00001eb310150 |
|  | MS.gene48278 |  | Os04g54790 |  | Zm00001eb272880 |
|  | MS.gene21118 |  | Os02g57160 |  | Zm00001eb144330 |
|  | MS.gene017355 |  | Os09g07660 |  | Zm00001eb365530 |
|  | MS.gene69233 |  | Os02g36570 |  | Zm00001eb340520 |
| Arabidopsis thaliana | AT4G31390 |  | Os07g12530 |  | Zm00001eb201710 |
|  | AT5G24970 |  | Os07g37180 |  | Zm00001eb216930 |
|  | AT1G79600 |  | Os04g56510 |  | |
